# In-Cell Protease Assay Systems Based on Trans-Localizing Molecular Beacon Proteins Using HCV Protease as a Model System

**DOI:** 10.1371/journal.pone.0059710

**Published:** 2013-03-21

**Authors:** Jeong Hee Kim, Min Jun Lee, Inhwan Hwang, Hyun Jin Hwang

**Affiliations:** 1 Department of Biochemistry and Molecular Biology, School of Dentistry, Kyung Hee University, Seoul, Korea; 2 Department of Life Science, POSTECH, Pohang, Korea; 3 R&D Center, Ahram Biosystems Inc., Seoul, Korea; University of Catania, Italy

## Abstract

This study describes a sensitive in-cell protease detection system that enables direct fluorescence detection of a target protease and its inhibition inside living cells. This live-cell imaging system provides a fluorescent molecular beacon protein comprised of an intracellular translocation signal sequence, a protease-specific cleavage sequence, and a fluorescent tag sequence(s). The molecular beacon protein is designed to change its intracellular localization upon cleavage by a target protease, i.e., from the cytosol to a subcellular organelle or from a subcellular organelle to the cytosol. Protease activity can be monitored at the single cell level, and accordingly the entire cell population expressing the protease can be accurately enumerated. The clear cellular change in fluorescence pattern makes this system an ideal tool for various life science and drug discovery research, including high throughput and high content screening applications.

## Introduction

Proteases are involved in many critical biological pathways and have been the focus of a broad range of biological and disease-related process studies, including apoptosis, Alzheimer’s disease and viral infections [1]–[4]. Inhibition or inactivation of a specific protease can inhibit or block cellular processes that are induced by that protease, and these proteases became attractive targets for drug development. Accordingly, there has been significant interest in developing new technologies for monitoring the activity of a target protease and its inhibition inside a living cell [5]–[6]. Compared to biochemical methods, cell-based assays have become an important part of the pre-clinical drug discovery process because cellular integrity and toxicity can be monitored at the time of performing the target assay. These advantages encourage development of cell-based assay for various targets in the cell [6]–[9]. The primary cell-based assays involved cell surface cutting or cell fixation [9]–[10] because developing technology that enables direct observation of live cells, and the targets within them, has been challenging. In this study, we developed in-cell protease assay systems based on molecular beacon reporter (MBR) proteins with intracellular trans-localizing properties that are modulated depending on the action or inaction of a target protease inside a living cell. The MBR proteins were designed to exhibit different trans-localization before and after a protease-induced cleavage. We demonstrate the effectiveness of these novel in-cell protease assay systems using the hepatitis C virus (HCV) NS3 protease and its cleavage sequence (CS) as a model system.

The MBR proteins used in this study were constructed to contain an NS3 serine protease-specific CS, an intracellular translocation signal sequence(s), and a fluorescent protein(s) to detect translocation of the fluorescent protein following NS3 protease-specific cleavage inside a living cell. Two types of MBR proteins were formulated: type I which shows translocation of a fluorescent protein from a subcellular organelle to the cytosol upon cleavage, and type II, which exhibits translocation of a fluorescent protein from the cytosol to a subcellular organelle upon cleavage. Using these MBR proteins, we have demonstrated that the mechanism and level of protease activity can be monitored at the single-cell level. Therefore, the level of the protease activity can be accurately enumerated in an entire cell population. Because the fluorescent image change of the cells can be clearly and easily monitored, this novel method is an ideal tool for biological and drug discovery researchers.

## Materials and Methods

### Cell Culture, Drug Treatment and Cytotoxicity Analysis

Chinese hamster ovary (CHO-K1, ATCC CCL-17) cells were cultured in appropriate media as recommended by the supplier. Exponentially growing cells were seeded at 5×10^5^ cells/well in a six-well plate and treated to test the in-cell protease assay systems as described in the Results section.

### Construction and Preparation of Plasmids

Plasmids encoding several substrate chimeric MBR proteins were constructed. For the construction of the type I substrate, green fluorescent protein (GFP) was amplified with primers 5′-GCG GGT ACC ATG GTG AGC AAG GGC GAG-3′ and 5′-GCG GAA TTC CTT GTA CAG CTC GTC CAT-3′ and cloned into the pcDNA3.1 vector (Invitrogen, USA). Subsequently, the proteolytic CS of the NS3 HCV protease was inserted between GFP and the Pleckstrin homology (PH) domain by polymerase chain reaction (PCR) amplification using primers 5′-GCG GAA TTC GAG GCC AAC GCG GAG GAT GTC GTG TGC TGC TCA ATG TCT TAC TCT TGG ACA GGC GCA CTC ATC GAT GAC TCG GGT AGG GAC TTC-3′ and 5′-GCG TCT AGA TCA CTG GAT GTT GAG CTC-3′. The resulting plasmid was named pHCV-CS1a/Ib-GFP.

For the type II substrate, two constructs, mono-color type II and dual-color type II were prepared. Glyceraldehydes-3-phosphate dehydrogenase (GAPDH) or GFP was used as a masking protein for mono-color or dual-color type II MBR proteins, respectively. To clone GAPDH, total RNA was prepared from CHO-K1 cells by using Trizol (MRC, USA) and cDNA was produced with reverse transcriptase (Invitrogen, USA). Complementary DNA for GAPDH was amplified with primers 5′-GCG GGT ACC ATG GGG AAG GTG AAG GTC GGA GTC-3′ and 5′-GCG GAA TTC CTC CTT GGA GGC CAT GTG GGC CAT-3′ and cloned into pcDNA3.1. Mitochondrial targeting sequence of methionine sulfoxide reductase (MSRA) was placed at the N-terminus of GFP by amplification using the primers 5′-GCG GAA TTC ATG CTC TCG GCC ACC CGG AGG GCT TGC CAG CTC CTC CTC CTC CAC AGC CTC TTT CCC GTC CCG AGG ATG GAC TCG GGT AGG GAC TTC-3′ and 5′-GCG TCT AGA TTA CTT GTA CAG CTC GTC CAT GCC-3′. The proteolytic CS of NS3 HCV protease was placed at the N-terminus of the mitochondrial targeting sequence by PCR-amplification using the primers 5′-GCG GAA TTC GAG GCC AAC GCG GAG GAT GTC GTG TGC TGC TCA ATG TCT TAC TCT TGG ACA GGC GCA CTC ATG CTC TCG GCC ACC CGG AGG-3′ and 5′-GCG GAA TTC GAG GAT GTC GTG TGC TGC TCA ATG TCT TAC ATG CTC TCG GCC ACC CGG AGG-3′ (CS:MSRA:GFP). Finally, the GAPDH sequence was placed at the N-terminus of the CS:MSRA:GFP clone without a stop codon to generate the plasmid, pHCV-CS1a/IIa-GFP.

To clone dual-color type II MBR (3.1-GFP:CS1a(10∶10):MSRA:RFP), the mitochondrial targeting sequence of MSRA was fused to RFP (MSRA:RFP) by PCR amplification with primers 5′-GCG GAA TTC ATG CTC TCG GCC ACC CGG AGG GCT TGC CAG CTC CTC CTC CTC CAC AGC CTC TTT CCC GTC CCG AGG ATG GTG CGC TCC TCC AAG AAC-3′ and 5′-GCG TCT AGA TTA CAG GAA CAG GTG GTG-3′. Next, the CS for HCV NS3 was placed in frame to generate CS:MSRA:RFP. For this purpose, primers 5′-GCG GAA TTC GAG GCC AAC GCG GAG GAT GTC GTG TGC TGC TCA ATG TCT TAC TCT TGG ACA GGC GCA CTC ATG CTC TCG GCC ACC CGG AGG-3′ And 5′-GCG TCT AGA TTA CAG GAA CAG GTG GTG-3′ were used. Finally, this construct was subcloned into a GFP containing vector at the *Eco* RI and *Xba* I sites. To generate plasmids expressing different sizes of CS, appropriate primers were prepared by the shortening above described primers to generate specified sequences, as described in the text. The HCV protease clone used in this study, pHCV-NS3/NS4A contained NS3 and NS4a domains for its enzymatic activity [11]–[13].

### Transfection and Observation of Intracellular the Protease Activity

Plasmid DNAs coding for the HCV NS3 protease and its substrate protein were prepared using the RPM turbo and/or maxiprep kits (Q-Biogene, USA) and transfected into 50% confluent CHO-K1 cells using the Gene Shuttle 40 kit (Q-Biogene, USA). This kit was also used to deliver the RNA aptamer into the cells for the inhibition tests. Cells were seeded into 12 well plates and transfected with plasmids coding for NS3 protease and its substrate MBR protein. The cells were observed under a fluorescent microscope (Nikon E800, Japan) at the appropriate time points after transfection. Cells exhibiting (i.e., ring-like shape for type I MBR or a dispersed pattern for type II MBR) or not exhibiting protease activity (i.e., dispersed pattern for type I MBR or speckles for type II MBR) were counted. At least three different experiments were performed for each assay and at least 500 cells were counted for each experiment. The relative percent cleavage was calculated as the number of cells exhibiting HCV protease inhibition divided by the total number of cells counted.

### Western Blot Analysis

After transfection, cell lysates were prepared as described previously [14] and the protein concentration was determined. After sodium dodecyl sulfate-polyacrylamide gel electrophoresis (SDS_PAGE), proteins were transferred to polyvinylidene difluoride (PVDF) membranes (Millipore, USA) for 2 hr at 80 mA. Blots were probed with a mouse monoclonal anti-GFP (BD Clontech, USA), Immunoreactivity was detected using either a peroxidase-conjugated anti-mouse igG (Santa Cruz Biotechnology, USA) or anti-rabbit IgG (Amersham, UK) peroxidase-conjugated secondary immunoglobulin G antibody followed by enhanced chemiluminescence kit (ECL, Amersham, UK). Experiments were repeated at least three times.

### Inhibition of HCV Protease

A known inhibitor of the HCV NS3 protease, Ac-Asp-D-Gla-Leu-Ile-b-cyclohexyl-Ala-Cyc-OH (N-1725.0001, Bachem, Germany) was purchased and dissolved in dimethyl sulfoxide. The stock solution was stored at –20°C and diluted in phosphate-buffered saline to the appropriate working concentration before addition to the cell culture medium during transfection process.

The HCV NS3 protease inhibitory RNA aptamer (G9-I) was prepared as described previously [15]. Briefly, we synthesized a single-stranded DNA template for the HCV NS 3 protease inhibitor template (NS3 I-temp), 5′-GGG AGA ATT CCG ACC AGA AGC TTC GGG ATT TGA GGG TAG AAT GGG ACT ACC TTT CCT CTC TCC TTC CTC TTC T-3′, using a DNA synthesizer (Expedite 8900 Nucleic Acid Synthesis System, Applied Biosystems, USA). This template was converted to a double stranded DNA molecule by DNA polymerization with the NS3 I-3′ primer 5′-AGA AGA GGA AGG AGA GAG GAA AGG-3′. The double stranded DNA was amplified by PCR using NS3 I-3′ and NS3 I-5′T7 (5′-AGT AAT ACG ACT CAC TAT AGG GAG AAT TCC GAC CAG AAG-3′) primers, purified with phenol, precipitated with ethanol and recovered by centrifugation. The RNA aptamer was produced from the PCR product by *in vitro* transcription using the RiboMAX kit (Promega, USA)., and added to cells at a concentration of approximately 7.5 µg/mL during transfection.and the cells were incubated until fluorescence image analysis.

## Results

### Construction of Plasmids Expressing MBR Proteins and their Subcellular Translocation Upon Cleavage by HCV Protease

**Figure 1:**
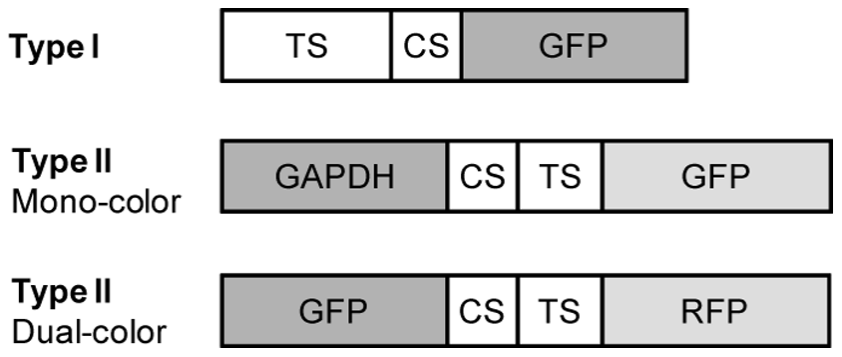
Schematic illustration of MBR proteins used in this study. One type I and two different type II MBRs were constructed. Either GAPDH or GFP was used as a masking protein for mono-color or dual-color type II MBR, respectively. TS; translocation signal, CS; cleavage sequence, GFP; green fluorescent protein, GAPDH; glyceraldehydes 3-phosphate dehydrogenase. RFP; red fluorescent protein.

MBR proteins used in this study are shown in Fig. 1. Two types of MBRs, type I and type II were constructed. Type I MBR contains an N-terminal PH domain as a translocation signal to the plasma membrane [16]–[17], a CS recognized by the HCV NS3 protease and GFP at the C-terminal. Thus the tagging fluorescent protein of type I MBR is designed to change its subcellular localization from a subcellular organelle, the plasma membrane, to the cytosol upon cleavage by HCV protease.

Type II MBRs contain a masking protein, CS, translocation signal and a fluorescent protein. Two different subtypes of type II MBR were generated; mono-color and dual-color. For the mono-color MBR, GAPDH was used as a masking protein and GFP was used as a fluorescent tag that changes its subcellular location upon cleavage by the protease. The mitochondrial targeting sequence of MSRA was used as a translocation signal [18]–[19] and was placed in between the masking protein and the tagging fluorescent protein. For the dual-color MBR, GFP was used as a masking protein and RFP was used as the tagging fluorescent protein. In this case, the tagging fluorescent protein was designed to re-distribute the type II MBR from the cytosol to another subcellular organelle, in this case, the mitochondria after cleavage by HCV protease.

**Figure 2:**
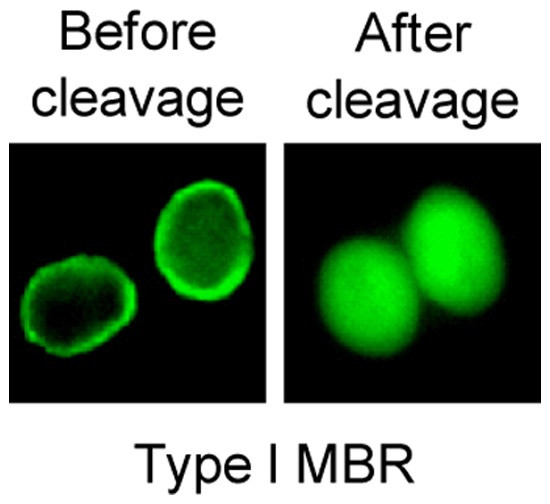
Detection of HCV NS3 protease using type I MBR. Before cleavage, GFP was found in the plasma membrane (ring shape, before cleavage). Cleavage of the type I MBR by HCV NS3 protease resulted in the translocation of GFP to the cytosol (dispersed image, after cleavage).

First, the expression and subcellular translocation of the MBRs created were observed. Since they contain a plasma membrane-targeting domain, type I MBR proteins and thus GFP, should be directed to the plasma membrane following transfection. As expected, GFP was observed in the plasma membrane as a ring-shape in living cells (Fig. 2, left, before cleavage). When type I MBR was co-expressed with HCV NS3 protease, GFP was observed in the cytosol in a dispersed pattern (Fig. 2, right, after cleavage). This result indicated that HCV NS3 protease cleaved the type I MBR, thereby changing the subcellular location of GFP from the plasma membrane to the cytosol. These changes in fluorescence localization were clearly observed without significant alterations to cell integrity.

**Figure 3:**
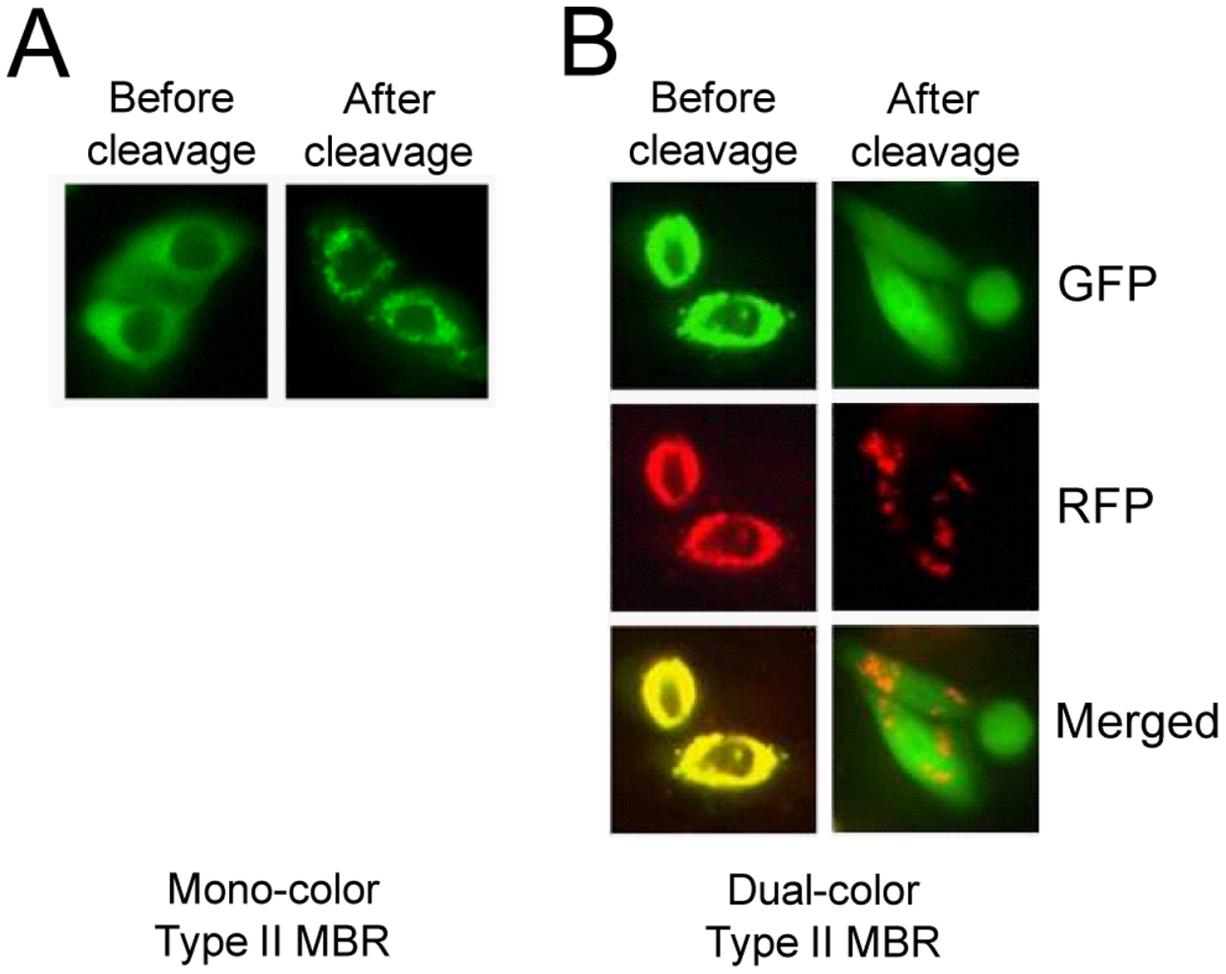
Detection of HCV NS3 protease using type II MBRs. Type II MBRs were constructed to contain a mitochondrial targeting sequence, MSRA as a masked translocation signal. The masking protein used was GAPDH or GFP for mono- or dual-color type II MBRs, respectively. **A**. Cleavage of mono-color type II MBR by HCV NS3 protease activates the mitochondrial translocation signal of MSRA, resulting in translocation of GFP from the cytosol (before cleavage) to the mitochondria (after cleavage). **B**. Cleavage of dual-color type II MBR which contains both GFP and RFP, results in translocation of the RFP from the cytosol (before cleavage) to mitochondria while GFP remains in the cytosol (after cleavage) following cleavage. GFP; green fluorescent protein, RFP; red fluorescent protein.

Next, we evaluated the fluorescence re-distribution of type II MBRs. The translocation signal of these proteins was blocked by a masking protein at the N-terminus. Before cleavage, mono-color type II MBR resides in the cytosol because the masking protein, GAPDH directs the localization of the MBR protein. Thus, GFP was dispersed throughout the cytosol (Fig. 3A, left, before cleavage). When it was cleaved by the co-expressed HCV NS3 protease, the mitochondrial targeting sequence was revealed at the N-terminus, thereby directing the MBR protein to the mitochondria. Thus, GFP localization changed to the mitochondria and it was observed as small speckles as expected (Fig. 3A, right, after cleavage). When dual-color type II MBR protein was expressed, MBR protein was expressed in the cytosol. Therefore, both GFP and RFP were observed in the cytosol as overlapping images. (Fig. 3B, left, before cleavage). Upon cleavage of dual-color type II MBR, the mitochondrial targeting sequence was revealed at the N-terminus, thereby targeting RFP to the mitochondria while maintaining GFP in the cytosol. As expected, RFP was observed in the mitochondria as small speckles and the green fluorescence was observed as a dispersed image in the cytosol (Fig. 3B, right, after cleavage). The fluorescence re-distribution of both mono- and dual-color type II MBRs was clearly observed in living cells with no significant effect on cell integrity. Similar results were obtained with different cell lines such as human embryonic kidney 293 cells (data not shown). These results demonstrate that the MBR proteins constructed in this study function exactly as designed and therefore can be used as a model assay system for HCV inhibitor screening.

### Comparison of Cleavage Sequences in MBR Proteins

**Figure 4:**
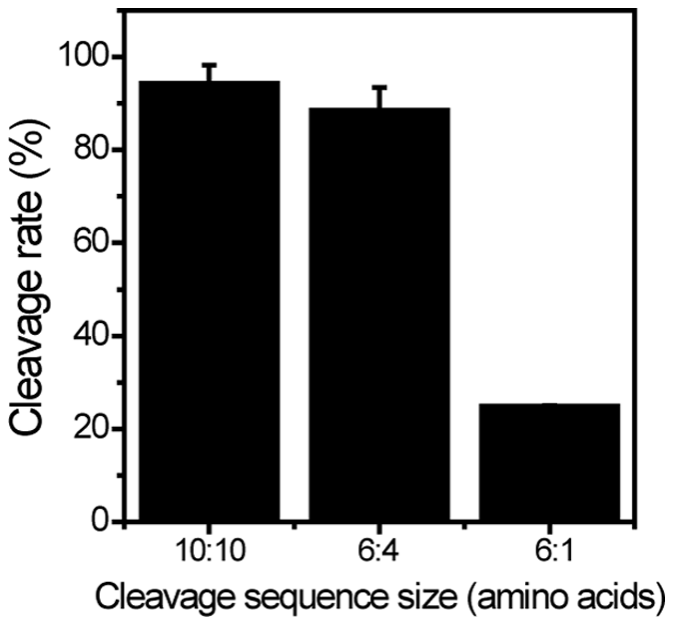
Cleavage sequence length-dependent cleavage efficiency of MBR protein. The cleavage sequence length examined were 20 (10 amino acids-cleavage site-10 amino acids), 10 (6 amino acids-cleavage site-4 amino acids), and 7 (6 amino acid-cleavage site-1 amino acid). Cleavage efficiency was measured using type I MBR and the data was plotted. Experiments were performed at least three times and the data is presented as mean ± standard deviation (SD).

The consensus sequence of the trans-cleavage sites recognized by HCV NS3 protease is D/EXXXXC-A/S, where X represents any amino acid and the scissile bond is located between C and A/S [9]. In order to optimize cleavage efficiency we compared the cleavage efficiency of the MBR constructs with various CS length, including EANAEDVVCC-SMSYSWTGAL (10∶10), EDVVCC-SMSY (6∶4), and. EDVVCC-S (6∶1). These three CS’s were designed to contain conserved amino acid sequences. Type I MBRs containing these three different CS’s were constructed and tested for cleavage efficiency. The number of cells with ring-shape fluorescence (uncleaved MBR) in the plasma membrane and cells with dispersed fluorescence in the cytosol (cleaved MBR) were counted to determine the MBR cleavage efficiency. As shown in Fig. 4, MBRs that contained 10 amino acids each before and after the CS (10∶10) as well as those that contained 6 amino acids before and 4 amino acids after the CS (6∶4) displayed higher cleavage efficiency at 94% and 89%, respectively. MBRs that contained 6 amino acids before and 1 amino acid after the CS (6∶1) showed approximately 25% cleavage. The same experiment was performed with type II MBRs and yielded similar results (data not shown). These results suggest that the optimum CS size with sufficient cleavage efficiency needs to be approximately equal to or greater than10 amino acids in length with conserved amino acid sequence. We used CS (6∶4) in MBR proteins from this point forward.

### Confirmation of MBR Protein Cleavage by Western Blot Analysis

The cleavage of MBR proteins was confirmed by Western blot analysis. Cells were transfected with vectors expressing either type I or type II MBRs. Another group of cells were co-transfected with vectors containing MBR proteins and HCV NS3 protease. Cells from both groups were harvested after the indicated period of incubation, and lysates were separated on SDS-PAGE, blotted onto PVDF membrane, and probed with anti-GFP antibody.

**Figure 5:**
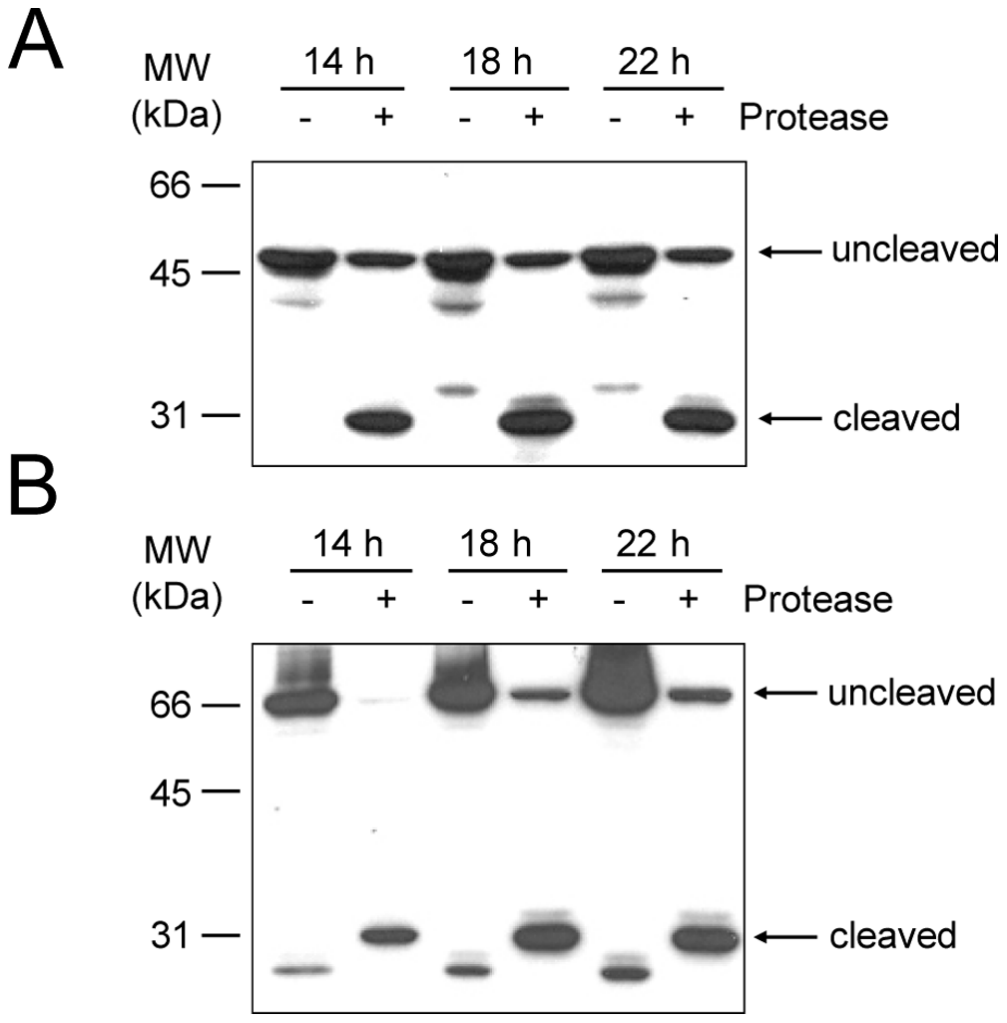
Western blot analysis of MBR protein cleavage. Type I (**A**) and type II (**B**) MBR proteins were expressed alone (−; lanes 1, 3, and 5 for A and B) or with protease (+; lanes 2, 4, and 6 for A and B). Cell lysates prepared after the indicated incubation times were subjected to immunoblotting with an anti-GFP antibody, as described in the Materials and Methods. **A**. The expected size of 50.2 kDa (uncleaved) and 27.4 kDa (cleaved) were observed with type I MBR protein. **B**. The expected size of 60.7 kDa (uncleaved) and 27.4 kDa (cleaved) were observed with mono-color type II MBR protein.

As shown in Fig. 5, the expressed MBR proteins were identified with the expected molecular weights. When MBR proteins were co-expressed with the HCV protease, the detectable MBR fragments were smaller in size as expected. Type I MBR was a 50.2 kDa protein (Fig. 5A, uncleaved) that upon cleavage, was reduced to a 27.4 kDa protein composed of GFP and a few amino acids from the cleavage sequence (Fig. 5A, cleaved). Protease cleavage was observed at 14, 18 and 22 hrs, with optimum observed after 18 hrs of incubation.

Cleavage of mono-color type II MBR was also tested by Western blot analysis. With GAPDH as a masking protein and GFP fused to the C-terminus (see Fig. 1), this construct generated a 67.0 kDa protein in the cell (Fig. 5B, uncleaved). After cleavage of the whole construct by HCV NS3 protease, the GAPDH masking protein is removed and GFP with the mitochondrial translocation signal at the N-terminus is generated. The N-terminal extension was lost upon transport into the mitochondria and GFP was detected in this organelle (Fig. 5B, cleaved). Once again, protease cleavage was at the experimental time points, 14, 18 and 22 hrs with effective cleavage observed at all time points.

### Protease Inhibition Analysis with Known Inhibitors

**Figure 6:**
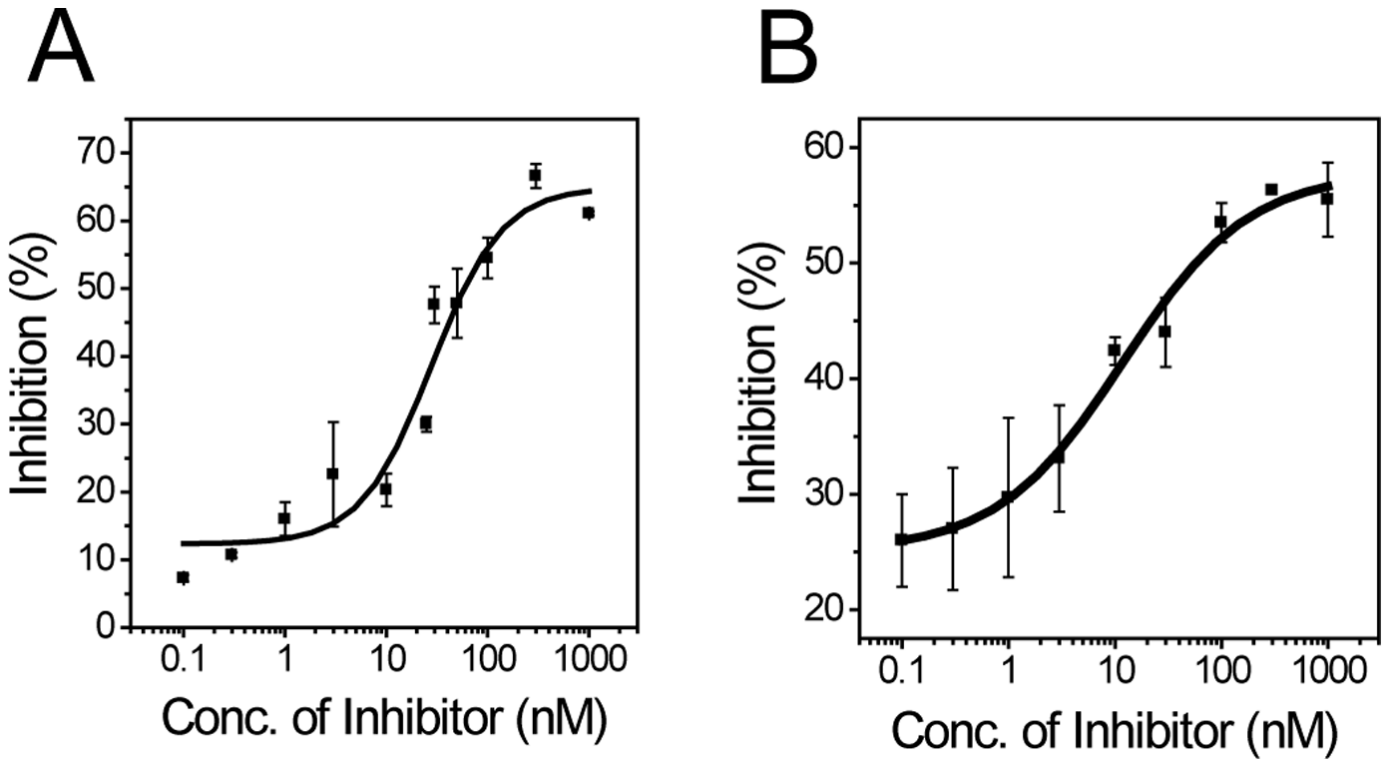
Inhibition of HCV NS3 protease by a known inhibitor. The cleavage of type I MBR (**A**) and type II MBR (**B**) by HCV NS3 protease was monitored in the presence of a known peptide derivative inhibitor, Ac-Asp-D-Gla-Leu-Ile-β-cyclohexyl-Ala-Cyc-OH. Both type I and type II MBRs exhibited significant dose-response at nanomolar concentrations.

Our in-cell protease assay system was validated with a known HCV inhibitory compound. A peptide derivative, Ac-Asp-D-Gla-Leu-Ile-β-cyclohexyl-Ala-Cyc-OH, which was reported as an HCV inhibitor [20] was used. The inhibitor was added to the cell culture medium during transfection and incubated with the cells as indicated. The number of cells with uncleaved and cleaved type I MBRs was counted by monitoring fluorescence localization changes inside living cells to obtain the MBR cleavage ratio at specific concentration of the protease inhibitor. As shown in Fig. 6A, a sigmoid curve was obtained. The half-maximal inhibitory concentration (IC_50_) value was estimated to be approximately 28±7 nM. A similar graph was obtained with type II MBRs (Fig. 6B). In this case, the number of cells with dispersed fluorescence in the cytosol and with speckle-like fluorescence in mitochondria was counted to generate dose-response curve. The IC_50_ value was approximately 12±6 nM. The values obtained with these two different MBRs overlap each other within a 2 sigma error. The reported *in vitro* IC_50_ value of the inhibitor used was 40 nM in 150 mM of NaCl [20]. The IC_50_ values achieved in this study were lower than the reported *in vitro* value. The NaCl concentration used in this study was approximately 100 mM.

**Figure 7:**
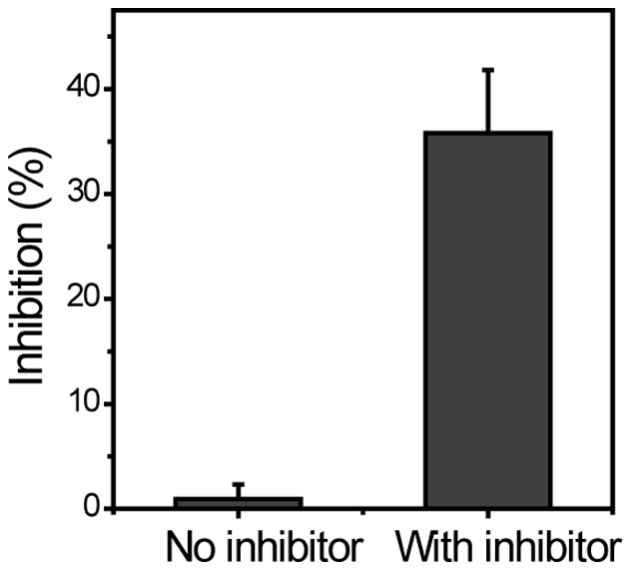
Inhibition of HCV NS3 protease by an RNA aptamer. An HCV NS3 protease inhibitory RNA aptamer was prepared and tested. Cells co-transfected with vectors encoding type I MBR and HCV NS3 protease were and treated with the RNA aptamer. The inhibition ratio was determined from the number of cells exhibiting a dispersed pattern relative to the total number of cells examined.

Inhibition of HCV NS3 protease was also examined with the in-cell-assay using a known RNA aptamer inhibitor [15]. Cells were co-transfected with vectors encoding type I MBR and HCV NS3 protease. Control cells were transfected with the MBR vector alone. The inhibitory RNA aptamer was added to the cell culture media during transfection at a concentration of approximately 7.5 µg/mL. Cleavage of the expressed type I MBR was detected by measuring the intracellular fluorescence distribution after a 14-hr incubation. As shown in Fig. 7, in the presence of inhibitory RNA aptamer, the inhibition rate was increased to approximately 35.8%. The inhibition rate was lower than the reported *in vitro* efficiency of 90%. However, considering drug delivery into the cell its intracellular stability, our results have significant meaning. We also found that the protease inhibiting activity was reduced over time (data not shown). These data imply that the RNA aptamer is degraded in the cell rapidly while the HCV protease was expressed continuously as the incubation time increases.

## Discussion

There is no vaccine against HCV available yet and the major standard treatment for HCV is combination treatment of pegylated interferon alpha with ribavirin [21]. A few HCV protease inhibitors are undergoing clinical trials [22]–[24]. However, resistance to the current HCV protease inhibitors cures has been reported [25]–[26]. These circumstances put more weight on the importance of developing HCV anti-viral drug candidates and a rapid, sensitive assay for the enzyme.

We developed an in-cell protease assay with HCV NS3 protease as a model system. We designed fluorescent molecular beacon-type reporters that allow the translocation of reporter proteins to be monitored from one cellular compartment to another in living cells by simply observing the re-distribution of fluorescence. The in-cell assay system developed in this study is capable of measuring protease activity in a single cell in an on/off mode and can be used for quantitative analysis of protease inhibitors. In addition, since this new assay is performed with living cells, cell integrity, drug delivery into the cell, possible toxicity of candidate compounds and other relevant information can be monitored, which is very advantageous. Therefore, the assay developed here can be applied to high-content screening assays.

To acquire more statistically accurate data from the in-cell assay, it is important to secure an appropriate number of cells that show a clear image of fluorescent re-distribution before and after cleavage. Factors, including transfection efficiency, expression of the MBRs, and incubation time before analysis of protease activity, are important and need to be considered [27]–[29]. We tried different transfection reagents, cell lines, translocation signals, expression vectors, and incubation times. To obtain statistically significant data, we counted more than 500 cells in each experiment and at least three independent experiments were performed for each assay. The data reported in this study was obtained using experimentally derived optimal conditions and results.

Another advantage of this assay is that various MBRs can be designed for assays to screen for inhibitors to a variety of proteases including other viral proteases, physiologically important proteases involved in other diseases, and signaling pathways. When applying the in-cell protease assay to other proteases, the minimum CS length recognized by the target protease can be identified and inserted into MBRs. We tested different CS lengths for HCV in this study and found that a CS with 10 amino acids, which is slightly longer than the reported consensus CS of 7 amino acids, yielded good cleavage [13]. It seems that space for effective protease binding is needed and must be considered when constructing MBRs for other proteases. Relatively longer CSs can be easily accommodated for in MBRs by a simple cloning process. Our new assay can also be applied to an HTS system. For additional applications, stable cell lines expressing an MBR and/or protease can be generated.

Along with its broad application possibilities, we demonstrated that this assay is easy and simple to perform and score the results. The cleavage efficiency of the MBRs developed in this study are highly sensitive, reaching approximately 90% cleavage. Therefore, this assay is very sensitive and can be applied to inhibitor screening without the need for complex statistical tools.
