# Correction: In-Cell Protease Assay Systems Based on Trans-Localizing Molecular Beacon Proteins Using HCV Protease as a Model System

**DOI:** 10.1371/annotation/2cdfa0af-64ff-4487-b1d8-34b56e63fe92

**Published:** 2013-04-05

**Authors:** Jeong Hee Kim, Min Jun Lee, Inhwan Hwang, Hyun Jin Hwang

There was an error in the Funding Statement. The correct Funding Statement is:

This study was supported by a grant from the Ministry of Health and Welfare (03-PJ1-PG11-VN01-SV02-0019 to HJH) and by the BIO & Medical Technology Development Program of National Research Foundation of Korea grant (MEST, 2012M3A9C6049936 to JHK). The funders had no role in study design, data collection and analysis, decision to publish, or preparation of the manuscript. 

